# Chemical characterization and extractives composition of heartwood and sapwood from *Quercus faginea*

**DOI:** 10.1371/journal.pone.0179268

**Published:** 2017-06-14

**Authors:** Isabel Miranda, Vicelina Sousa, Joana Ferreira, Helena Pereira

**Affiliations:** Centro de Estudos Florestais, Instituto Superior de Agronomia, Universidade de Lisboa, Lisboa, Portugal; University of British Columbia, CANADA

## Abstract

Heartwood and sapwood of *Quercus faginea* were evaluated in relation to summative chemical composition and non-polar and polar extracts composition, including an assessment of antioxidant properties (DPPH and FRAP). Twenty trees from two sites in Portugal were analysed. Heartwood had approximately two times more solvent extractible compounds than sapwood (on average 19.0% and 9.5%). The lipophilic extractible compounds were below 1%, and most of them were polar e.g. ethanol-soluble compounds corresponded to 65% of total extractives in heartwood and 43% in sapwood. Lignin content was similar in sapwood and heartwood (28.1% and 28.6% of extractive-free wood respectively) as well as the sugar composition. Site did not influence the chemical composition. The lipophilic extractible compounds from both sapwood and heartwood included mainly saturated fatty acids (23.0% and 36.9% respectively) and aromatic compounds were also abundant in sapwood (22.9%). The ethanol-water extractibles had a high content of phenolic substances (558.0 and 319.4 mg GAE/g extract, respectively of heartwood and sapwood). The polyphenolic composition was similar in heartwood and sapwood with higher content of ellagitannins (168.9 and 153.5 mg tannic acid/g of extract in sapwood and heartwood respectively) and very low content of condensed tannins. The antioxidant activity was very high with IC_50_ of 2.6 μg/ml and 3.3 μg/ml for sapwood and heartwood respectively, as compared to standard antioxidants (IC_50_ of 3.8 μg/ml for Trolox). The ferric reducing ability was 2.8 and 2.0 mMol Trolox equivalents/g extract of heartwood and sapwood respectively. The variability between trees was low and no differences between the two sites were found. *Q*. *faginea* showed a very good potential for cooperage and other applications for which a source of compounds with antioxidant properties is desirable.

## Introduction

The Portuguese oak (*Quercus faginea* Lam., identified by A. Fabião) is a species from *Fagaceae* family, native to the Western Iberian Peninsula and the North African countries of Morocco, Tunisia, and Algeria. It is a medium-sized deciduous or semi-evergreen tree growing to a height of 20 m and a diameter of 80 cm. This species occupied once (XV-XVI centuries) much of the Portuguese territory and the wood was valued and intensively exploited for naval construction [1]. However, the large stands of *Q*. *faginea* trees are now reduced to a few scattered stands, especially in mixed stands with other oaks. This decline occurred mostly in the last three decades of the twentieth century as a consequence of land use change from forest to agriculture and to a preferential reforestation with other species e.g. *Q*. *suber*, *Q*. *rotundifolia*, *Eucalyptus globulus* and *Pinus pinaster* [1]. Today the oak forests in Portugal are mostly of *Q*. *suber* (23% of the total forest area), and *Q*. *rotundifolia* (11%) while the other oak stands occupy about 2% of the forest area with *Q*. *faginea* restricted to only a few scattered stands [2].

The wood of *Q*. *faginea* is not presently used in a significant extent. However, the wood potential and the environmental and cultural importance of the species are acknowledged. General descriptions of *Q*. *faginea* wood refer good aesthetic appearance, white yellowish sapwood and brown yellowish heartwood, high density, and considerable mechanical strength [3, 4]. Recent research efforts have been made to increase knowledge on growth and wood characteristics with the objective of contributing to value *Q*. *faginea* for high-quality end-uses, thereby strengthening the species sustainability. Results already obtained include ring analysis and heartwood development [5], wood anatomy [6] and density [4, 7] and their relationship [8]. The enological potential of *Q*. *faginea* heartwood was also recently considered, showing a balanced tannins content, mainly of hydrolysable tannins such as ellagitannins, of volatile phenols and phenolic acids, in levels similar to or greater than those shown by the American (*Q*. *alba*) or French (*Q*. *petraea*) oaks [9–14].

A chemical characterization of *Q*. *faginea* has not been made, namely considering the differences between heartwood and sapwood. In fact the transformation of sapwood to heartwood is accompanied by accumulation of solvent extractible compounds (the so-called extractives) and their proportion and properties may have a significant impact on the wood utilization. For instance, the heartwood in oaks shows high concentrations of phenolic extractibles (up to 10%), that may have multiple biological effects, including antioxidant properties, and has small amounts of lipids that vary according to the species and other factors such as geographical origin and tree [10, 12, 15–17].

In this study, the summative chemical composition of the heartwood and sapwood of twenty *Q*. *faginea* trees from two sites in Portugal was evaluated, and the content and composition of lipophilic and polar extractible compounds were determined. The underlying rationale is an evaluation of the chemical-related wood quality of *Q*. *faginea* and of its variability for value wood products that could enhance the exploitation of this endogenous forest species, also taking into account the potential relevance of its wood extractives.

## Material and methods

The study was carried out in two locations: one stand in the northeast of Portugal (site 1), near Macedo de Cavaleiros and the other stand in the centre of Portugal (site 2), near Vimeiro. For the first site, the authority who issued the permission was Instituto da Conservação da Natureza e das Florestas ICNF, and for site 2 the private owner was asked and gave permission.

### Site characterization and sampling

A total of twenty *Q*. *faginea* trees were randomly selected and harvested from two naturally regenerated and unmanaged stands (10 trees per stand) within the natural geographic distribution area of the species: one stand with 34–60 year old trees in the northeast of Portugal (site 1), near Macedo de Cavaleiros (41° 30′ N, 7° 01′ W; 554 m altitude); the other stand with 112–150 year old trees in the centre of Portugal (site 2), near Vimeiro (39° 29′ N, 9° 01′ W; 100 m altitude). The climate is of the Mediterranean type with Atlantic influence with a mean annual temperature of 12°C and 15°C, and annual precipitation of 700 mm and 890 mm at site 1 and site 2, respectively. Soils are classified as leptosols at site 1 and cambisols at site 2 [6]. The tree characteristics are summarised in Table 1.

**Table 1:** Characteristics of the sampled *Quercus faginea*.

	Site 1	Site 2
Tree height (m)	10.5 ± 0.7^a^	14.8 ± 2.3^b^
Diameter (cm)*	20.9 ± 4.2^a^	26.7 ± 5.9^b^
Tree age*	40 ± 8^a^	125 ± 11^b^
Sapwood width (mm)	37.9 ± 15.5^a^	23.1± 5.8^b^
Heartwood (% total area)	37.1 ± 15.9^a^	73.1 ± 4.6^b^

*Diameter (Including bark) measured at 1.3m of tree height and age based in ring counts at the stem base.

Mean of ten trees and standard deviation. Means with the same letter in one line are not significantly different.

The chemical determinations were performed on a stem disc collected at breast height with approximately 5 cm thickness. The sapwood and heartwood were separated with a chisel and the samples were ground in a knife mill (Retsch SM 200) and sieved (Retsch ISO 9001). The 40–60 mesh fractions were kept for analysis.

### Chemical analysis

Chemical summative analysis included determination of ash, soluble extractible compounds in dichloromethane, ethanol and water, Klason and acid-soluble lignin, and the monomeric composition of polysaccharides. All determinations were made with duplicate samples.

The ash content was determined by incinerating 2.0 g of the sample at 525°C overnight and weighing the residue, reported as percent of the original samples (TAPPI 211 om-93). The soluble extractible compounds were determined with procedures adapted from Tappi 204 cm-97, in a soxhlet system with dichloromethane, ethanol and water during 6 h, 16 h and 16 h respectively. The extractible compounds solubilised by each solvent were determined by mass difference of the solid residue after drying at 105°C and reported as percent of the original sample (TAPPI T204 om-88). The lignin content was analysed from the extracted samples by acid hydrolysis with 72% sulphuric acid. Klason lignin was determined as the mass of the solid residue after drying at 105°C (TAPPI T 222 om-02). The acid-soluble lignin was determined by analysing the UV absorbance at 206 nm using a UV/VIS spectrophotometer (TAPPI Useful Method UM 250). The remaining acid solution was kept for sugar analysis.

The polysaccharides were calculated based on the amount of the neutral sugar monomers released by total hydrolysis, after derivatization as alditol acetates and separated by high-performance anion exchange chromatography (Aa Dionex ICS-3000 system equipped with an electrochemical detector). The separation was performed with Aminotrap plus CarboPac SA10/Carbopac PA10 anion-exchange columns. The mobile phase was an aqueous 2 nM NaOH 135 solution at a flow rate of 1.0 ml/min at 25°C.

### Composition of dichloromethane extracts

The lipophilic extractible compounds that were solubilized from the wood samples with dichloromethane were recovered as a solid residue after evaporation of the solvent and dried overnight under vacuum at room temperature. Aliquots (2 mg) of each sample were taken and derivatized as described below. They were dissolved in 100 μL of pyridine and the compounds with hydroxyl and carboxyl groups were trimethylsilylated into trimethylsilyl (TMS) ethers and esters, respectively, by adding 100 μl of bis(trimethylsily)-trifluoroacetamide (BSTFA). The reaction mixture was heated at 60°C for 30 min in an oven.

The derivatized extracts were immediately analyzed by GC—MS by injection in a GC—MS Agilent 5973 MSD with the following GC conditions: Zebron 7HG-G015-02 column (30 m, 0.25 mm; ID, 0.1 μm film thickness), flow 1 ml/min, injector 280°C, oven temperature program, 100°C (1 min), rate of 10°C/min up to 150°C, rate of 4°C/min up to 300°C, rate of 5°C/min up to 370°C, rate of 8°C/min up to 380°C (5 min). The MS source was kept at 220°C and the electron impact mass spectra (EIMS) taken at 70 eV of energy.

Compounds were identified as TMS derivatives by comparing their mass spectra with a GC—MS spectral library (Wiley, NIST), and by comparing their fragmentation profiles with published data [18, 19]. The full scan of the chromatogram was used in order to find all possible compounds. For semi-quantitative analysis the area of peaks in the total ion chromatograms of the GC—MS analysis was integrated and their relative proportions expressed as area proportion of the total chromatogram area. Each aliquot was injected in triplicate and results presented by mean (only standard deviation inferior to 5% was considered).

### Composition of ethanol-water extracts

Extracts were prepared using approximately 1 g of the wood samples and ethanol/water (50/50, v/v), with a 1:10 (m/v) solid-liquid ratio for 60 min at 50°C using an ultrasonic bath. After filtration the supernatant extract was used to determine the contents in total phenolics, and condensed and hydrolysable tannins. Each assay was performed at least three times and at least three independent replicates were prepared for each standard and sample.

The total phenolic content was determined spectrophotometrically by the Folin—Ciocalteu method using gallic acid as standard [20]. An aliquot (100 μL) of the extract was mixed with 4 ml of the Folin—Ciocalteu reagent and after 6 min, 4 ml of a 7% Na_2_CO_3_ solution was added. After 15 min of incubation in a bath at 45°C, absorbance was read at 760 nm versus a prepared blank. A calibration curve was built using gallic acid as a standard (0–150 μg/ml). The total phenolic content was expressed as mg of gallic acid equivalents (GAE) per g of extract.

Condensed tannins were determined by the vanillin-H_2_SO_4_ method, and the results expressed as mg of (+)-catechin equivalents [21]. An aliquot (1.0 ml) of the extract was mixed with 2.5 ml of 1.0% (m/v) vanillin in absolute methanol and then with 2.5 ml of 25% (v/v) sulphuric acid in absolute methanol for vanillin reaction with the polyphenols in the extract. The blank solution was prepared with the same procedure without vanillin. Absorbances were recorded at 500 nm after 15 min. The tannin content was calculated from a calibration curve using catechin as standard, and expressed as mg of catechin equivalents (CE) per g of the extract.

Hydrolyzable tannins were determined by the method of Willis and Allen [22]. One ml of the extract and 5 ml of 2.5% KIO_3_ were added into a vial and vortexed for 10 s. Absorbance of the red coloured mixture was determined at 550 nm versus the prepared water blank. Six concentrations of tannic acid solutions (500–2000 mg/L) were used for calibration. The final results were expressed as mg tannic acid equivalent (TAE) per g of the extract.

### Antioxidant activity of ethanol-water extracts

Two methods were used to determine the antioxidant properties of these samples: ferric reducing/antioxidant power (FRAP), which measures the sample’s ferric reducing power, and 2,2-diphenyl-1-picryhydrazyl (DPPH), which measures the free radical scavenging capacity.

The DPPH assay was performed using 2,2-diphenyl-1-picrylhydrazyl hydrate (DPPH) [23] and was expressed in terms of: a) the amount of extract required to reduce 50% of the DPPH concentration (IC_50_); and b) Trolox equivalents on a dry extract base. First, different dilutions of the initial extract and of a Trolox solution (0.2 mg/ml) in methanol were prepared. An aliquot of 100 μL of each methanolic solution of extract and of Trolox were added to 3.9 ml of a DPPH methanolic solution (24 μg/ml). The blank sample consisted of 100 μl of methanol added to 3.9 ml of DPPH solution. After 30 min incubation at room temperature in the dark, the absorbance was measured at 515 nm.

The radical scavenging activity of each sample was calculated by the DPPH inhibition percentage as follows: I % = [(Abs_0_-Abs_1_)/Abs_0_]×100, where Abs_0_ was the absorbance of the blank and Abs_*1*_ was the absorbance in the presence of the extract at different concentrations.

The IC_50_ inhibiting concentration, which represents the concentration of a sample necessary to sequester 50% of the DPPH radicals, was obtained by plotting the inhibition percentage against the extract concentration. The scavenging effect on the DPPH radical of the extract was also expressed as the Trolox equivalent antioxidant capacity (TEAC) calculated from the calibration curve with the Trolox solution concentrations and the percentage of scavenging effect on the DPPH radical.

The ferric reducing antioxidant power (FRAP) assay was followed as described by [24]. The FRAP reagent was generated by mixing 300 mM sodium acetate buffer (pH 3.6), 10 mM (tripyridyl triazine) TPTZ solution and 20.0 mM FeCl_3_.6H_2_O solution in a ratio of 10:1:1 in volume. A sample (100 μl) of extract or standard was then added to 3 ml of FRAP reagent and the reaction mixture was incubated at 37°C for 30 min. The absorbance was measured at 593 nm in comparison with a blank. Aqueous solutions of known Trolox concentrations in the range of 0–0.5 Mmol/L were used for the calibration curve, and the results were expressed as Mmol Trolox equivalents/ g dry mass.

### Statistical analysis

All results were expressed as mean and standard deviation (SD). The significance of differences (*p* ≤ 0.05) among the corresponding mean values was determined using one-way analysis of variance (ANOVA) followed by Duncan’s multiple-comparison test, and the Student’s t-test, Shapiro-Wilk normality test was also made using *Sigmaplot*^®^ statistical software, version 11.0.

## Results and discussion

The heartwood is clearly distinct in the wood cross-section, showing a rich brown colour and well defined border to the sapwood. The studied trees differ between sites by age, diameter and heartwood proportion as shown in Table 1: site 2 has the older and larger trees, therefore with a higher heartwood content (73.1% vs. 37.1% at site 2 and site 1).

### Chemical composition

The chemical composition of the heartwood and sapwood of the *Q*. *faginea* trees from the two sites is given in Tables 2 and 3 regarding the monosaccharide composition.

**Table 2:** Summative chemical composition (% of total dry mass) of the sapwood and heartwood of *Quercus faginea*.

	Site 1		Site 2	
	Sapwood	Heartwood	Sapwood	Heartwood
Ash	1.0 ± 0.4	0.5 ± 0.2	0.8 ± 0.2	0.4 ± 0.2
Extractives Total	10.8 ± 2.4^a^	19.3 ± 2.7^b^	8.2 ± 1.6^c^	18.8 ± 2.8^b^
Dichloromethane	0.6 ± 0.2^a^	0.7 ± 0.1^b^	0.6 ± 0.2^a^	0.9 ± 0.3^c^
Ethanol	5.9 ± 1.8^a^	13.2 ± 1.9^b^	2.6 ± 0.9^c^	11.5 ± 3.2^b^
Water	4.3 ± 0.8^a^	5.4 ± 0.9^b^	5.0 ± 0.9^a^	6.4 ± 0.9^c^
Lignin Total	25.1 ±1.1^a,b^	23.7 ± 0.9^b^	25.8 ± 1.5^a^	22.6 ± 1.1^c^
Klason lignin	22.3 ± 1.3^a^	21.0 ± 1.0^b^	22.9 ± 1.5^a^	20.3 ± 1.1^b^
Soluble lignin	2.8 ± 0.3^a^	2.7 ± 0.4^a^	2.9 ±0.1^a^	2.3 ± 0.3^b^
Polysaccharides	55.7 ± 3.7^a^	50.2 ± 2.8^b^	57.3 ± 2.1^a^	54.0 ± 3.2^c^

Mean of ten trees and standard deviation. Means with the same letter in one line are not significantly different.

**Table 3:** Monosaccharide composition of sapwood and heartwood of *Quercus faginea*, in % of total neutral monosaccharides.

	Site 1		Site 2	
	Sapwood	Heartwood	Sapwood	Heartwood
Rhamnose	0.6 ± 0.06^a^	0.55 ± 0.04^a^	0.6 ± 0.1^a^	0.53 ± 0.1^a^
Arabinose	2.8 ± 0.4^a^	2.7 ± 0.5^a^	2.3 ± 0.3^c^	5.3 ± 3.6^c^
Xylose	31.4 ± 1.2^a^	30.3 ± 0.8^b^	30.6 ± 1.7^c^	27.7 ± 1.7^c^
Mannose	3.6 ± 1.3^a^	4.2 ± 0.9^a^	3.1 ± 0.5^a^	3.7 ± 1.1^a^
Galactose	2.2 ± 0.4^a^	2.4 ± 0.3^a^	2.2 ± 0.4^a^	2.4 ± 0.7^a^
Glucose	59.3 ± 1.5^a^	59.9 ± 1.5^b^	61.2 ± 2.1^c^	60.4 ± 2.8^a^

Mean of ten trees and standard deviation. Means with the same letter in one line are not significantly different.

There were considerable chemical differences between heartwood and sapwood as regards solvent extractible compounds content (Table 2). Heartwood contained significantly higher (F (1, 18) = 142.72; *p <* 0.001) amounts of extractible compounds than sapwood: approximately two times more with on average 19.0% and 9.5% in heartwood and sapwood respectively.

Polar compounds extracted by ethanol and water, which include mainly phenolics and polyphenolics, corresponded to most of the total content (93% and 96% of the total extractible compounds in sapwood and heartwood respectively). However, there was a difference between the proportion of the ethanol and water soluble compounds in heartwood ethanol-soluble compounds represented the largest proportion of total extractives (65%) while in sapwood it was the water-soluble extractibles that had the highest proportion (50% of the total extractives). The amount of dichloromethane soluble extractibles was very small (below 1%) similar in heartwood and sapwood, but statistically different (F (3, 36) = 6.60; *p* = 0.001) (Table 2).

Comparative studies of heartwood—sapwood solvent extractible compounds in other oak species have shown in general a heartwood enrichment in these non-structural compounds, although to a variable extent depending on species. Seikel et al. [25] reported that *Q*. *rubra* contained 6.2% and 8.2% total extractibles in sapwood and heartwood, respectively, which included sixteen phenolic compounds. Hernández and Salazar [26] reported the chemical composition of sapwood and heartwood of four oak species: respectively 6.1% and 7.2% in *Q*. *coccolobilofia*, 8.3% and 8.9% in *Q*. *durifolia*, 9.2% and 11.6% in *Q*. *rugose*, and 8.2% and 10.6% in *Q*. *oleoides*. Ruiz-Aquino et al. [27] found no differences between sapwood and heartwood solvent extractible compounds content: respectively 5.3% and 5.4% in *Q*. *laurina*, and 8.2% and 8.9% in Q. *crassifolia*.

The higher content in solvent extractible compounds of heartwood in relation to sapwood is considered a heartwood specific feature [28]. However the magnitude of the difference between sapwood and heartwood differs greatly between species e.g. respectively 3.5% and 5.7% or 2.4% and 3.8% in *Eucalyptus globulus* [29, 30], 4.0–4.2% and 7.4–9.5% in *Acacia melanoxylon* [31], 9.2% and 10.0% in *Tectona grandis* [32].

The heartwood contained slightly less lignin than sapwood (23.1% and 25.5% in relation to wood, Table 2) but when calculated in relation to extractive-free wood the lignin content is the same: 28.6% in heartwood and 28.1% in sapwood.

The lignin content reported for *Q*. *faginea* heartwood and sapwood is similar to the values reported by Ruiz-Aquino et al. [27] for *Q*. *laurina* (25.1% in sapwood and 25.5% in heartwood) and *Q*. *crassifolia* (24.9% in sapwood and 25.2% in heartwood), and by Lourenço et al. [33] for *Q*. *suber* sapwood (23.8%).

The carbohydrate composition was similar in heartwood and sapwood (Table 3). The major monosaccharide was glucose (60.8% and 59.6% of the total monomeric sugars in heartwood in sapwood respectively) while xylose was the dominating non-cellulosic sugar (31.6% and 33.8% of total neutral monosaccharides in heartwood ad sapwood respectively) and arabinose and galactose represented about 2.9% of the total content of neutral sugars. This sugar composition agrees with the general structure of hardwood hemicelluloses [34]. A similar monosaccharide composition was reported for *Q*. *suber* sapwood [33].

Overall there was little chemical variation between the *Q*. *faginea* trees and the sapwood and heartwood chemical composition was not influenced by the sites (and therefore by the different ages of trees) e.g. *p* = 0.131 for heartwood solvent extractible compounds, *p* = 0.153 for extractive-free heartwood lignin content.

### Composition of dichloromethane extract

The identified lipophilic extractible compounds in *Q*. *faginea* sapwood and heartwood are reported in Table 4. The main constituents are saturated alkanoic acids (15.7% and 25.8% in sapwood and heartwood, respectively). The chain lengths vary from C10 to C26, with hexadecanoic acid as the major compound found, representing 35.5% and 41.1% of all fatty acids present in sapwood and heartwood, respectively. Both saturated and substituted alkanoic acids are substantially more abundant in heartwood than in sapwood as previously reported by Ekman [35], although there is significant less content of substituted fatty acids, probably due to side reactions that convert them into the saturated form [28]. Sterols were also abundant in both wood tissues, varying from 10.2% to 13.0%. Among them, β-sitosterol constitutes 6.6% to 12.1% of the identified compounds. Fatty acids and sterols are responsible for the reduction of wood durability [36] since they are nutrients for fungi attack. However, antifungal capacity was found for some fatty acids, as reported by Salem et al. [37].

**Table 4:** Composition of dichloromethane extracts of *Quercus faginea* samples, in % of the chromatographic peak areas of the compounds detected by GC-MS.

Identified Compounds	Sapwood			Heartwood	
	Site 1		Site 2	Site 1	Site 2
Aromatic compounds	22.48±0.60	23.16±0.64		5.28±0.39	5.26±0.95
4-Hydroxy-3-methoxybenzaldehyde (vanillin)	0.88±0.03	0.86±0.02		0.39±0.04	0.39±0.08
1,4-Dihydroxy-2,6-dimethoxybenzene	0.15±0.01	0.18±0.01		-	-
4-Hydroxy-3,5-dimethoxybenzaldehyde (syringaldehyde)	1.10±0.02	1.09±0.06		0.54±0.02	0.55±0.10
4-Hydroxy-3-methoxybenzoic acid (vanillic acid)	3.98±0.08	3.97±0.10		0.26±0.03	0.26±0.06
3-Hydroxy-4-methoxybenzoic acid (isovanillic acid)	-	-		1.07±0.00	1.08±0.07
3-(4-Hydroxy-3,5-dimethoxyphenyl)prop-2-enal (Sinapaldehyde)	0.68±0.02	0.68±0.06		0.09±0.00	0.10±0.02
3-(4-hydroxy-3,5-dimethoxyphenyl)prop-2-enoic acid (Sinapic acid)	0.17±0.02	0.17±0.01		-	-
4-Hydroxy-3-methoxyphenyl glycol	2.87±0.03	2.86±0.03		-	-
3-Hydroxy-4-methoxyphenyl glycol	0.79±0.01	0.81±0.03		-	-
δ-Tocopherol	0.50±0.02	0.50±0.08		-	-
4-Hydroxy-3,5-dimethoxybenzoic acid (syringic acid)	2.31±0.07	2.30±0.04		0.88±0.08	0.90±0.24
3-(4-hydroxy-3-methoxy-phenyl)prop-2-enoic acid (ferulic acid)	1.34±0.04	1.37±0.01		0.10±0.03	0.10±0.03
3-Hydroxy-1-(4-hydroxy-3-methoxyphenyl)propan-1-one	1.96±0.03	1.93±0.03		0.52±0.09	0.54±0.11
2-Hydroxy-1-(4-hydroxy-3,5-dimethoxyphenyl)ethan-1-one	1.63±0.01	1.47±0.05		0.17±0.03	0.09±0.02
2,3-Dihydroxy-1-(4-hydroxy-3-methoxyphenyl)propan-1-one	3.14±0.08	4.12±0.08		0.56±0.05	0.55±0.12
1-(4-hydroxy-3,5-dimethoxyphenyl)ethan-1,2-diol	0.50±0.10	0.49±0.02		0.70±0.02	0.70±0.10
3-Hydroxy-1-(4-hydroxy-3,5-dimethoxyphenyl)propan-1-one	0.8±0.03	0.36±0.01		-	-
Alkanols	3.04±0.26	3.30±0.14		4.40±0.18	4.53±0.83
1-Hexadecanol	0.14±0.01	0.14±0.06		-	-
1-Octadecanol	0.32±0.00	0.46±0.01		-	-
1-Docosanol	1.16±0.08	1.24±0.02		2.45±0.07	2.31±0.51
1-Tricosanol	0.33±0.06	0.32±0.01		-	-
1-Tetracosanol	0.61±0.04	0.85±0.00		1.29±0.02	1.29±0.26
1-Pentacosanol	0.31±0.01	0.13±0.01		-	-
1-Hexacosanol	0.17±0.02	0.16±0.03		0.10±0.02	0.36±0.05
1-Eicosanol	-	-		0.19±0.04	0.19±0.00
22-Methyltricosanol	-	-		0.37±0.03	0.38±0.01
Saturated alkanoic acids	14.75±0.56	16.61±0.26		25.64±3.85	25.87±3.25
Decanoic acid	-	-		0.25±0.04	0.25±0.05
Dodecanoic acid	0.51±0.04	0.41±0.01		0.16±0.01	0.22±0.00
Tetradecanoic acid	1.23±0.09	1.25±0.05		0.30±0.07	0.29±0.04
Pentadecanoic acid	1.59±0.01	1.59±0.02		-	-
Hexadecanoic acid	7.21±0.14	9.15±0.02		15.15±2.03	15.21±1.22
Heptadecanoic acid	0.63±0.07	0.71±0.04		-	-
Octadecanoic acid	1.43±0.04	1.37±0.04		0.75±0.05	0.71±0.16
Eicosanoic acid	0.14±0.00	0.13±0.00		-	-
Docosanoic acid	0.29±0.01	0.29±0.01		3.58±0.54	3.54±0.24
Tricosanoic acid	0.10±0.01	0.14±0.02		-	-
Tetracosanoic acid	1.19±0.07	1.15±0.06		3.10±0.67	3.26±1.01
Hexacosanoic acid	0.43±0.08	0.42±0.01		2.35±0.44	2.39±0.53
ɑ-Hydroxy alkanoic acids	-	-		0.70±0.21	0.84±0.11
24-Hydroxytetracosanoic acid	-	-		0.70±0.21	0.84±0.11
Substituted alkanoic acids	4.78±0.05	4.91±0.09		10.49±1.25	10.42±1.13
9-*cis*-Hexadecenoic acid	-	-		0.13±0.03	0.09±0.03
9,12-Octadecadienoic acid	1.49±0.01	1.65±0.02		3.01±0.05	3.55±0.06
11-*cis*-octadecenoic acid	2.65±0.01	2.62±0.03		6.69±1.13	6.13±0.98
13-*cis*-octadecenoic acid	0.33±0.02	0.33±0.02		0.66±0.04	0.65±0.06
10-Nonadecenoic acid	0.31±0.01	0.31±0.02		-	-
Saturated diacids	1.29±0.04	1.52±0.04		0.45±0.11	0.49±0.05
Nonandioic acid (azelaic acid)	0.60±0.02	0.63±0.02		0.14±0.05	0.19±0.04
Decanedioic acid	0.69±0.02	0.89±0.02		-	-
Hexadecanedioic acid	-	-		0.31±0.06	0.30±0.01
Substituted diacids	1.00±0.09	1.12±0.06		0.25±0.07	0.23±0.03
2-Hydroxydecanedioic acid	0.20±0.06	0.34±0.03		0.25±0.07	0.23±0.03
2-Butenedioic acid	0.12±0.01	0.11±0.01		-	-
2-hexylpropanedioic acid	0.68±0.02	0.67±0.02		-	-
Sterols	11.76±0.29	9.35±0.13		12.83±2.39	12.75±2.80
Campesterol	0.25±0.03	0.24±0.02		-	-
Stigmasterol	1.11±0.01	1.14±0.00		0.30±0.09	0.26±0.00
β-Sitosterol	6.39±0.11	6.81±0.09		12.10±2.26	12.06±2.74
Stigmastanol	1.92±0.08	1.16±0.02		0.43±0.01	0.43±0.06
Stigmasterol	0.69±0.02	-		-	-
γ-Sitostenone	1.19±0.03	-		-	-
Stigmastane-3,6-dione	0.21±0.01	-		-	-
Glycerol derivatives	1.69±0.04	2.17±0.06		6.74±0.71	6.92±2.16
Glycerol	-	-		0.39±0.06	0.51±0.11
2-Hexadecanoylglycerol	0.23±0.00	0.13±0.02		0.19±0.02	0.11±0.05
1-Hexadecanoylglycerol	1.46±0.04	2.04±0.04		1.64±0.04	1.70±0.45
1-Octadecanoylglycerol	-	-		0.29±0.07	0.29±0.11
Monoacylglycerol C22	-	-		0.27±0.00	0.21±0.03
Monoacylglycerol C24	-	-		2.49±0.37	2.57±0.87
Monoacylglycerol C26	-	-		1.47±0.15	1.53±0.54
Triterpenes/Triterpenoids	5.38±0.25	5.23±0.41		15.29±3.28	10.78±2.32
β-Amyrin	0.73±0.05	0.63±0.16		0.21±0.03	0.26±0.06
Betulinic acid	0.70±0.03	0.35±0.01		-	-
Lupeol	0.42±0.02	0.41±0.03		0.51±0.11	0.05±0.02
Betulin	0.30±0.02	0.20±0.03		-	-
Oleanolic acid	0.65±0.01	0.73±0.05		-	-
Hederangenin	0.38±0.03	0.33±0.04		-	-
Corosolic acid	0.86±0.01	1.12±0.07		-	-
Arjunic acid	1.34±0.08	1.46±0.02		14.57±3.14	10.47±2.24
Other	16.65±0.48	16.77±1.37		17.66±0.35	17.48±3.17
3-Nonen-1-ol	1.79±0.08	1.65±0.09		-	-
3,4-Dihydroxybutanoic acid	-	-		0.05±0.02	0.06±0.01
Sugars	2.38±0.21	2.74±0.12		0.60±0.21	0.65±0.02
Sitosteryl-3β-glucopiranoside	12.48±0.19	12.38±1.16		17.01±3.11	16.77±3.14
Identified	82.82	84.14		97.68	95.57
Non-identified	17.18	15.86		2.32	4.43
Total	100	100		100	100

Mean of triplicate injection from the dichloromethane extracts of ten trees and standard deviation.

Triterpenes were also identified in both wood tissues, being higher in heartwood; arjunic acid was the major constituent (2.4% and 12.5% in sapwood and heartwood, respectively). This can be explained by the tree defence mechanism against external aggressors as fungi, accumulating triterpenes, sterols, flavonoids and other secondary metabolites during heartwood formation [38].

Authors found a significant positive relationship between the compositions of the lipophilic extractibles on both sapwood and heartwood tissues (p<0.05) concerning alkanols, alkanoic acids, sterols and triterpenes, from both sites (Table 5). The same is true for all the classes of compounds from both wood tissues from both sites, except for saturated and substituted diacids.

**Table 5:** Ethanolic aqueous extraction of sapwood and heartwood of *Quercus faginea*.

	Site 1		Site 2	
	Sapwood	Heartwood	Sapwood	Heartwood
Extraction yield (%)	7.9 ± 2.2^a^	14.9 ± 1.8^b^	4.0 ± 1.1^c^	14.4 ± 2.5^b^
Total phenolic (mg GAE/g extract)	346.3 ± 66.4^a^	581.2 ± 35.4^b^	292.8 ± 97.7^a^	534.7 ± 20.4^b^
Total phenolic (mg GAE/g wood)	27.1 ± 6.9	86.2 ± 9.7	11.9 ± 6.5	77.4 ± 12.7
Tannins (mg CE/g extract)	6.1 ± 2.0^a^	3.5 ± 2.3^a,c^	4.9 ± 1.6^b^	3.6 ± 2.3^c^
Hydrolysable tannins (mg TAE/g extract)	166.1 ± 24.9^a^	155.1 ± 16.3^b^	171.7 ± 68.9^b^	151.9 ± 34.8^a^
Hydrolysable tannins (mg TAE/g wood)	12.0 ± 5.6	22.8 ± 1.8	12.2 ± 7.4	24.0 ± 8.9
Antioxidant capacity (mg TEAC/g extract)	1303.2 ± 256.6	777.5 ± 96.8	1870.7 ± 579.2	769.9 ± 92.5
Antioxidant capacity (mg TEAC/g wood)	100.2 ± 17.2	114.1 ± 0.9	73.1 ± 29.7	111.4 ± 8.6
IC_50_ values (μg extract / ml)*	2.85 ± 0.7	3.10 ± 0.6	2.4 ± 1.32	3.6 ± 0.4
FRAP (mM TEAC/g extract)	2.1 ± 0.3	2.8 ± 1.2	1.9 ± 0.4	2.8 ± 0.3
FRAP (mM TEAC/g wood)	0.2 ± 0.03	0.40 ± 0.2	0.12 ± 0.2	0.40 ± 0.05

* IC_50_ Trolox in ethanol-water 3.81 μg Trolox/ml; GAE—gallic acid equivalents; CE—catechin equivalents; TAE tannic acid equivalent; TEAC—Trolox equivalent antioxidant capacity.

Mean of ten trees and standard deviation. Means with the same letter in one line are not significantly different.

The major difference between sapwood and heartwood lipophilic extracts relies on their aromatics content, reaching in average for the two sites 22.8% of all compounds for sapwood, against 5.3% in heartwood.

### Ethanol-water extracts

Table 5 shows the average and standard deviation values for the yield and composition of ethanol-water (1:1) extracts regarding total phenolics, condensed and hydrolysable tannins.

The results show a clear difference between heartwood and sapwood. The obtained extraction yield was high (on average 14.7% and 6.0% for heartwood and sapwood); these values are under the determined total solvent extractible compounds content (Table 2) due to the less intensive extraction conditions. However the obtained extract yields were higher in comparison to the value of 8.9% reported for *Q*. *faginea* heartwood extraction with 50% methanol-water or the values of 8.3%, 7.8% and 7.9% for *Q*. *pyrenaica*, *Q*. *robur* and *Q*.*petraea* respectively [10].

The major part of solvent extractibles is made up of phenolic substances (Table 5) that represent about 56% of the heartwood extract and 32% of the sapwood extract, corresponding to about 8% of heartwood (86.2 and 77.4 mg GAE /g of wood) and 2.5% of sapwood (27.1 and 11.9 mg GAE /g of wood). It is clear that there is an enrichment in phenolic compounds in the heartwood in comparison to sapwood. The values obtained here for the phenolic content of *Q*. *faginea* heartwood extracts are similar to those reported in the literature (no reports on sapwood extract composition were found). Cadahía et al. [12] and Fernandez de Simón et al. [10] reported for *Q*. *faginea* heartwood methanolic extracts (50% methanol-water) total phenolics of 50.5 and 89.2 mg GAE/g wood, respectively, and 42.6 and 56.6 mg GAE/g of wood for *Q robur*, 53.7 and 70.3 mg GAE/g of wood for *Q*. *petraea*, and 44.8 and 56.6 mg GAE/g of wood for *Q*. *pyrenaica* heartwood. Alañón et al. [39] obtained for methanol extracts values of 41.5 mg GAE/g of wood for *Q*. *pyrenaica*, 72.6, 48.9 and 33.3 mg GAE/g of wood for *Q*. *robur*, *Q*. *petraea* and *Q*. *alba* respectively.

The polyphenolic composition of the aqueous ethanolic extracts of *Q*. *faginea* heartwood and sapwood showed a similar profile, with a higher content in hydrolysable tannins (ellagitannins) and very small amounts of condensed tannins. This is in accordance with the literature [10, 12, 15, 17]. When expressing the amounts of hydrolysable tannins as mg tannic acid/g of wood, there is a difference between extracts due to the different extraction yields with higher values for the heartwood (on average 23 mg tannic acid/g of heartwood) in relation to sapwood (12.2 mg of tannic acid/g of sapwood). These values are higher than those reported by Fernandez de Simón et al. [10] and Cadahía et al. [12] who showed for *Q*. *faginea* an ellagitannin content of 4.0 and 8.5 mg ellagic acid/g wood. The values are also higher when compared to the 5.1–5.9 mg ellagic acid/g heartwood of *Q*. *pyrenaica*, 8.5–12.1 mg ellagic acid/g heartwood of *Q*.*petraea* and 7.6–8.2 mg ellagic acid/g heartwood of *Q*. *robur* [10, 12]. However, Castro-Vázquez et al. [40] report higher values for *Q*. *pyrenaica* with 18.2–23.0 mg ellagic acid/g wood.

The site did not influence the content of total phenolics of *Q*. *faginea* wood (F (1, 18) = 3.18; *p* = 0.080). The same was reported for *Q*. *pyrenaica* heartwood [41, 42, 43]. However for *Q*. *alba* and *Q*. *robur*, Miller et al. [44] referred that species seem to be more important than the geographical origin to explain the differences in the wood phenolic composition.

### Antioxidant properties of ethanol-water extracts

The antioxidant characteristics of *Q*. *faginea* heartwood and sapwood extracts were evaluated by measurement of the scavenging capacity against a given radical (DPPH assay) and by the ferric reducing antioxidant power (FRAP). The results are summarised in Table 5.

Both heartwood and sapwood extracts exhibited high antioxidant activities (IC_50_ of 2.6 μg/ml and 3.3 μg/ml respectively) when compared to Trolox (IC_50_ 3.8 μg/ml) which is used as an antioxidant standard compound. Ellagitannins should be the main compounds responsible for the antioxidant capacity of the extracts since they have a higher ability to donate a hydrogen atom and to support the unpaired electron as compared to low molecular weight phenolic compounds [39].

The antioxidant activity expressed as mg of Trolox equivalents/g of wood (112.7 mg Trolox equivalents/g of heartwood vs. 84.8 mg Trolox equivalents/g of sapwood) are similar to those reported for wood extracts of other oaks: 65.3 mg Trolox equivalents /g of wood and 112.9 mg Trolox equivalents /g of wood for American and French oaks respectively [43].

The heartwood and sapwood ethanolic extracts present FRAP activity with a mean value of 2.8 mMol Trolox/g extracts of heartwood and 2.1 mMol Trolox/g extracts of sapwood. The reducing ability of the extracts showed an increasing trend with the polyphenol content, as expected. Phenolic compounds can act as reducing agents, hydrogen donors, singlet oxygen quenchers or metal chelators due to their redox properties and consequently antioxidant capacity. The heartwood extract, with the highest concentration, exhibited the highest reducing capability (0.4 mMol Trolox equivalents/g of heartwood), while the sapwood extract possessed the lowest ferric reducing abilities (0.15 mMol Trolox equivalents/g of sapwood). The present results were compared to the values of other extracts from wood of *Quercus* spp. Alañón et al. [45] evaluated the FRAP of aqueous methanolic extract of *Q*. *pyrenaica*, *Q*. *robur*, *Q*. *petraea* and *Q*. *alba* in mMol of Trolox equivalents/g of wood as 0.54, 0.82, 0.59 and 0.39 respectively. In methanol extracts of American and French oaks, the FRAP values were 0.29 and 0.45 mMol Trolox equivalents/g of wood, respectively [39].

Overall the chemical features of the polar extracts of heartwood and sapwood of *Q*. *faginea* allow considering their potential use for the maturation and aging of alcoholic beverages, as previously suggested [10–14]. Also the use of extracts as a source of compounds with antioxidant properties may be envisaged as additives in the various applications for which such characteristics may be important.

## Conclusions

The heartwood and sapwood of *Q*. *faginea* are clearly outsingled with a well-established border. Chemically, heartwood and sapwood differ considerably in their extractives content, namely in polar extractives e.g. compounds soluble in ethanol and water, which are approximately two-fold in heartwood in relation to sapwood. The ethanol-water extracts are rich in phenolics, especially the heartwood extracts. The content of ellagitannins is high and of condensed tannins is low and similar in both heartwood and sapwood extracts. The antioxidant properties of *Q*. *faginea* heartwood and sapwood extracts are excellent and above those of standard antioxidant compounds.

Lipophilic extracts from *Q*. *faginea* heartwood and sapwood are mainly constituted by saturated alkanoic acids and sterols. Aromatic compounds were also identified in considerable extent only in the sapwood lipophilic extracts.

The variability between trees is low and no differences between trees of different ages from two sites were found.

*Q*. *faginea* shows a very good potential for cooperage and other applications for which a source of compounds with antioxidant properties is desirable.
